# A Steep Increase in the HeartLogic Index Predicts COVID-19 Disease in an Advanced Heart Failure Patient

**DOI:** 10.1155/2020/8896152

**Published:** 2020-07-11

**Authors:** Ward Heggermont, Pham Anh Hong Nguyen, Chirik-Wah Lau, Kurt Tournoy

**Affiliations:** ^1^Cardiovascular Research Center, Department of Cardiology, OLV Ziekenhuis Aalst-Asse-Ninove, Aalst, Belgium; ^2^Cardiovascular Research Institute Maastricht, Maastricht, Netherlands; ^3^Department of Respiratory Medicine, OLV Ziekenhuis Aalst-Asse-Ninove, Aalst, Belgium; ^4^Faculty of Medicine and Life Sciences, Ghent University, Ghent, Belgium

## Abstract

We present a patient with severe nonischemic cardiomyopathy in whom the HeartLogic algorithm was activated on her Boston Scientific cardioverter defibrillator. She had an out-of-alert state for several months and had clinically “stable” heart failure with no hospitalizations in the last 6 months. A sudden and fast increase of the HeartLogic index preceded her presentation in the emergency ward by several days. The detailed readout of HeartLogic however had some atypical features for heart failure decompensation. The patient presented at the emergency department with an increased dyspnea and a dry cough. Clinical exam showed desaturation and was suggestive for an acute respiratory infection. Subsequent imaging with CT thorax and nasopharyngeal real-time polymerase chain reaction (RT-PCR) confirmed SARS-CoV-2 viral pneumonia (COVID-19). This case illustrates that a timely and detailed analysis of HeartLogic alerts could help in the early differentiation of disease in patients with severe heart failure.

## 1. Introduction

The COVID-19 pandemic, caused by SARS-Coronavirus-2 (SARS-CoV-2), is rapidly changing our daily clinical practice around the globe [1]. Due to restrictions in normal clinical practice and the reservation of crucial hospital and physician's capacity to treat critically ill COVID-19 patients, a shift from routine ambulatory follow-up consultations to focused patient contacts based on disease severity, telemedicine, and device-based monitoring is being observed [2].

Remote monitoring of patients with internal cardioverter defibrillators (ICDs) and cardiac resynchronization therapy (CRT-pacing or CRT-defibrillator) is already common these days [3]. However, these devices also increasingly allow for device-based telemonitoring of the patient's disease state, especially for heart failure. Older examples of these kinds of applications are OptiVol and CorVue [4, 5]. Recently, HeartLogic emerged as a potent predictor of impending heart failure decompensation [6–11].

HeartLogic (HL) is a multiparametric algorithm that combines different device-based features (first and third heart sounds, thoracic impedance, respiratory rate, tidal volume, tidal volume variation, heart rate, and physical activity) into a single alert number. A predefined threshold, standard set at 16, defines whether the patient is in “cardiac homeostasis”—usually referred to as an euvolemic state in heart failure (index below 16), versus whether an impending heart failure episode is about to happen (index of 16 or more). However, HL holds promise to be useful for the early detection of alternative diagnoses besides cardiac decompensation. Here, we present a case in which a sudden and steep increase of HeartLogic with atypical features of heart failure decompensation increased the suspicion for an alternative diagnosis and helped in the early detection of a severe COVID-19 pneumonia. To date, this is the first case in which COVID-19 disease is revealed by the individual HeartLogic parameters.

## 2. Case Presentation

We present the case of a 72-year-old woman (nonsmoker) of African origin. In 2017, an idiopathic nonischemic dilated cardiomyopathy was diagnosed by means of echocardiography, with a left ventricular ejection fraction (LVEF) of 25%. Subsequent left and right heart catheterization revealed normal coronary arteries. The only known cardiovascular risk factor she had at the time of diagnosis was arterial hypertension which was treated accordingly. Magnetic resonance imaging revealed strong suspicion of left ventricular noncompaction and confirmed the low LVEF of 28%. Genetic analysis revealed no abnormalities. A two-chamber ICD was implanted in primary prevention. It was on this ICD (Boston Scientific G447 LV-plugged CRT-D) that the HeartLogic feature was enabled since June 2018.

The patient was stable in NYHA class II for more than 6 months under the following heart failure therapy: spironolactone 50 mg uid, bumetanide 1 mg bid, bisoprolol 2.5 mg bid, and sacubitril valsartan 49/51 mg bid. She also takes L-thyroxin 0.1 mg uid, thiamazol 10 mg uid (for hyperthyroidism), pantoprazole 40 mg uid, acetylsalicylic acid 80 mg uid, and simvastatin 20 mg uid.

**Figure 1:**
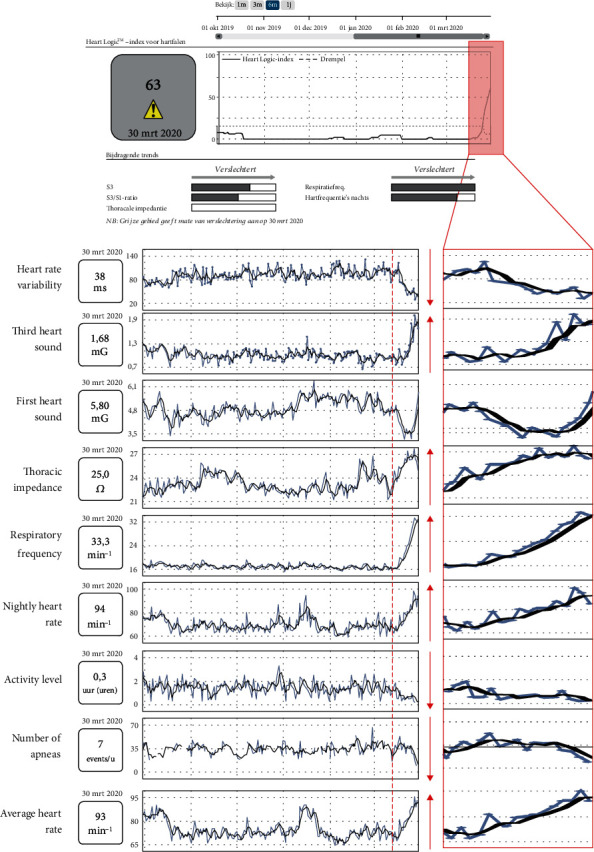
Overview of HeartLogic general evolution and contributing trends. Here, the general evolution of HL parameters is shown with a rapid and steep increase in the last seven days. It is observed that the third heart sound (S3), respiratory frequency, and nightly heart rate contribute to the increased HL index. Also, surprisingly, thoracic impedance rapidly increases. In contrast, heart rate variability and activity level significantly decreased. Also, due to the increased respiratory frequency, the number of apneas decreased.

Concordant with the euvolemic state, the HeartLogic (HL) index was consistently under the threshold level for more than 6 months (Figure 1). Seven days before admission, however, the HL index started to rise extremely fast, starting from a value of 9 till a HL index of 63 on the day of presentation at the emergency department. (Figure 1, Supplemental Figure (available here). The parameters that contributed to the out of hospital alert were a decreased heart rate variability, a strongly increased third heart sound, a rapidly changing first heart sound (i.e., a strong decrease followed by a steep increase), an increased thoracic impedance, an increased respiratory rate, an increased nightly heart rate, and a lower activity level. Concomitantly, a decreased number of apneas and an average increased heart rate were observed.

This patient presented at the emergency department with severe dyspnea. The dyspnea symptoms started 36 hours before admission. A few days prior, the patient suffered from a dry cough. She could not recall to having experienced fever; no fever was measured upon admission (36.2 degrees Celsius). She had not been visiting COVID-19-risk regions, but a close neighbor was diagnosed with SARS-CoV-2. Clinical exam revealed the known mitral insufficiency murmur as well as a normal pulmonary auscultation without wheezes or rhonchi. Respiratory rate was 25-30/min. Blood pressure was 137/80 mmHg with a pulse of 70 bpm. Peripheral oxygen saturation was 92% in ambient air. Electrocardiography showed a sinus rhythm of 70/min with signs of left ventricular hypertrophy and a QRS width of 110 ms, identical to previous ECGs. Blood analysis showed slight leucopenia, absolute lymphocyte count of 930 mcg/L, D-dimer of 4701 ng/L, LDH 301 IU/L, a C-reactive protein of 70 mg/L and an acute chronic renal insufficiency with an estimated GFR of 50 mL/min (normally 70 mL/min) (Table 1).

**Figure 2:**
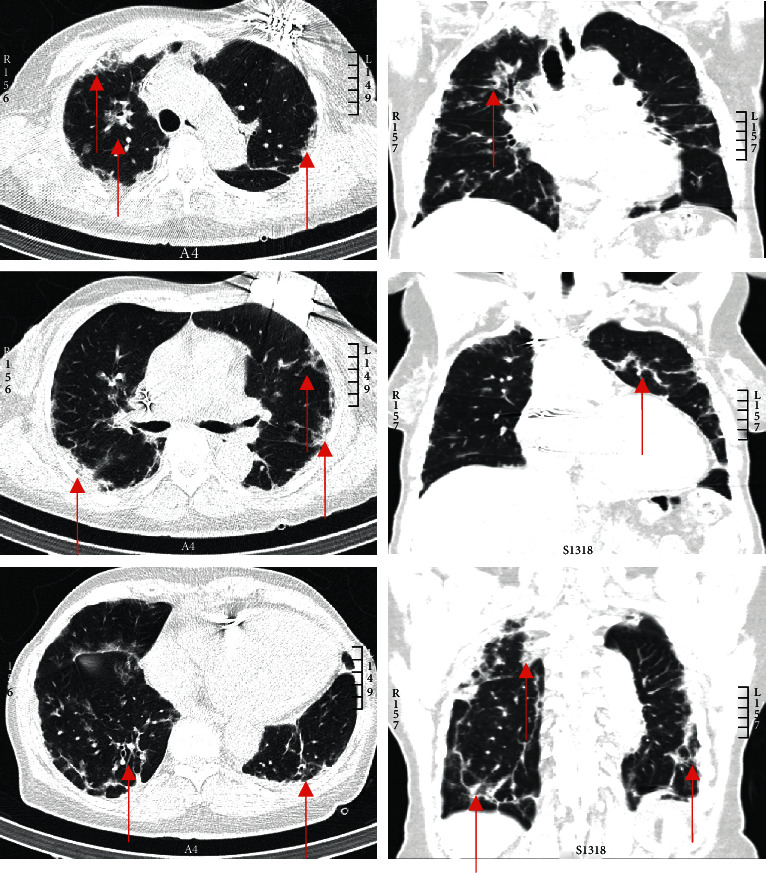
Overview of thoracic CT-scan revealing important COVID-19 disease. The arrows show the bilateral multifocal subpleural ground glass opacities, peribronchovascular infiltrates with partial consolidation, and thin pleural strands in both lower lobes.

A chest CT scan without contrast was performed to diagnose and assess the lung involvement since the high suspicion of COVID-19. The CT scan revealed bilateral multifocal subpleural ground glass opacities, with peribronchovascular distribution and partial consolidation, and thin pleural strands in both lower lobes. No pulmonary edema and no pleural effusion were observed (Figure 2). A nasopharyngeal swab confirmed the presence of SARS-CoV-2 by means of PCR assay; the patient was subsequently hospitalized in a dedicated COVID-19 ward with the required isolation measures.

## 3. Discussion

This is a timely case report wherein device-based telemonitoring delivered a warning signal that was atypical for heart failure alone, holding the promise to help in the early diagnosis of other health problems such as a viral pneumonia, here caused by SARS-CoV-2. Device-based telemonitoring is rapidly emerging as an interesting tool for remote follow-up in heart failure patients with a pacemaker, ICD, or CRT device [3]. Also, in other branches of medicine (wireless follow-up of glycaemia in diabetic patients, follow-up of therapeutic adherence to continuous positive airway pressure therapy in obstructive sleep apnea [12, 13]), remote patient follow-up allows for faster processing of specific patient data to control larger patient cohorts. These features rapidly evolved from “gadgets” to useful tools, especially when overall health care system capacity is stretched to its maximum, as is the case with this COVID-19 pandemic.

The HeartLogic device helps in the early detection of heart failure. Based on the validation study [6], the median time between the rise-above-threshold of HL and the clinical event was more than 30 days, allowing for corrective measures in order to prevent heart failure hospitalizations. Two large studies currently investigate the broader clinical applicability of HeartLogic: the MANAGE-HF study (ClinicalTrials.gov Identifier: NCT03237858) which tests the clinical usefulness of HeartLogic to prevent heart failure and related mortality and the PREEMPT-HF study (ClinicalTrials.gov Identifier: NCT03579641) which tests the clinical usefulness of HeartLogic to detect other (non-HF-related) meaningful clinical problems.

This case report supports this idea and hints at the possibility to make a differential diagnosis of dyspnea in heart failure patients using the HL device during the current SARS-CoV-2 pandemic. The rapidly increasing respiratory frequency together with an *increasing* thoracic impedance pleads against a pure cardiac decompensation with subsequent fluid overload and hints towards an important alternative diagnosis, a hypothesis that was corroborated by the CT scan findings and finally by showing SARS-CoV-2 in the nasopharyngeal swab. Indeed, the parameters that contributed significantly to the alert helped to differentiate COVID-19 from heart failure exacerbation. We observed an *increased* thoracic impedance, whereas normally a lower impedance would be expected for severe HF decompensation [14], as well as an out-of-proportion increase in respiratory frequency (Figure 1). As a matter of fact, thoracic impedance is generally calculated by measuring the energy needed to send a small current from the battery of a device (the “extrathoracic component”) to an intracardiac lead (the “intrathoracic component”). If lung alveoli are progressively filled up with water (pulmonary edema due to decompensation), resistance decreases as the conductivity for electric current is higher for water than for air [15]. Therefore, an increased thoracic impedance is very atypical for cardiac decompensation. In this case, the increase in thoracic impedance might be explained by multiple factors, e.g., strongly increased respiratory frequency and air trapping due to hyperinflation.

Also, in many cases with established heart failure, an increased heart rate is explained by atrial fibrillation whereas here a sinus tachycardia was noted. In addition, the overall speed of changes increased the suspicion of an alternative diagnosis. It is however important to emphasize that the infection with SARS-CoV-2 will have contributed to worsening heart failure. Indeed, respiratory infections are often sufficient to flip a euvolemic HF patient towards decompensation and fluid overload. Of course, the HL algorithm cannot replace the regular clinical evaluation but should be seen as an interesting help to raise a timely suspicion for the presence of an alternative diagnosis for heart failure or at least a diagnosis that can trigger cardiac decompensation.

An important issue is how to respond timely to the “early” changes detected by HL. Especially during this SARS-CoV-2 pandemic, hospital services are being reorganized for a full focus on the control of this infection. However, the permanent vigilance for aberrant “typical” or “atypical” alert patterns on the home-monitoring devices in these patients with heart failure might hold the promise for the early detection of a diagnosis of cardiac origin and beyond. In this case, it was a potentially lethal viral infection with SARS-CoV-2.

## 4. Conclusion

Device-based telemonitoring of heart failure patients can help to detect noncardiac diseases that significantly alter overall homeostasis. Moreover, detailed analysis of the HeartLogic algorithm can help to shed light on the diagnosis. To the best of our knowledge, this is the first case report in which a sudden increase in HeartLogic preceded a diagnosis of COVID-19.

## Figures and Tables

**Table 1:** Overview of blood work parameters and comparison with reference blood work upon last ambulatory cardiology consultation. The last outpatient visit was six months before the admission to the emergency ward, and the patient was in NYHA class II heart failure without signs of decompensation at that time.

Parameter	Unit	Emergency ward	Last consultation	Reference value
WBC count	Number/mcL	3520	4250	4000-10000
Lymphocytes	Number/mcL	930	—	1200-3600
D-dimer	mcg/L	4607	—	<500
Hemoglobin	g/dL	11.8	10.9	12-16
INR	—	1.2	1.0	0.8-1.2
NT-pro-BNP	ng/L	2503	1757	<125
LDH	IU/L	301	—	135-250
CRP	mg/L	70.6	8.5	<5.0
Glucose	mg/dL	98	—	<100
Creatinine	mg/dL	1.06	0.75	<0.90
eGFR	mL/min	51	76	>90
Sodium	mmol/L	137.6	142.6	135-145
Potassium	mmol/L	4.4	4.24	3.5-4.9

## Data Availability

This statement is not applicable as it is a case report and all relevant data are already available in the case report.

## Supplementary Materials

Supplementary Materials Supplemental Figure: overview of numeric HeartLogic parameters and contributing parameters. Detailed numeric evolution of the HeartLogic index and the individual contributors.
