# Supplementary material for: A Steep Increase in the HeartLogic Index Predicts COVID-19 Disease in an Advanced Heart Failure Patient
Source: Case Rep Cardiol. 2020 Jul 11;2020:8896152. doi: 10.1155/2020/8896152 (PMC7354669; doi:10.1155/2020/8896152)
Supplement: Supplementary Materials — Supplemental Figure: overview of numeric HeartLogic parameters and contributing parameters. Detailed numeric evolution of the HeartLogic index and the individual contributors. [file 8896152.f1.pptx]

## Slide 1
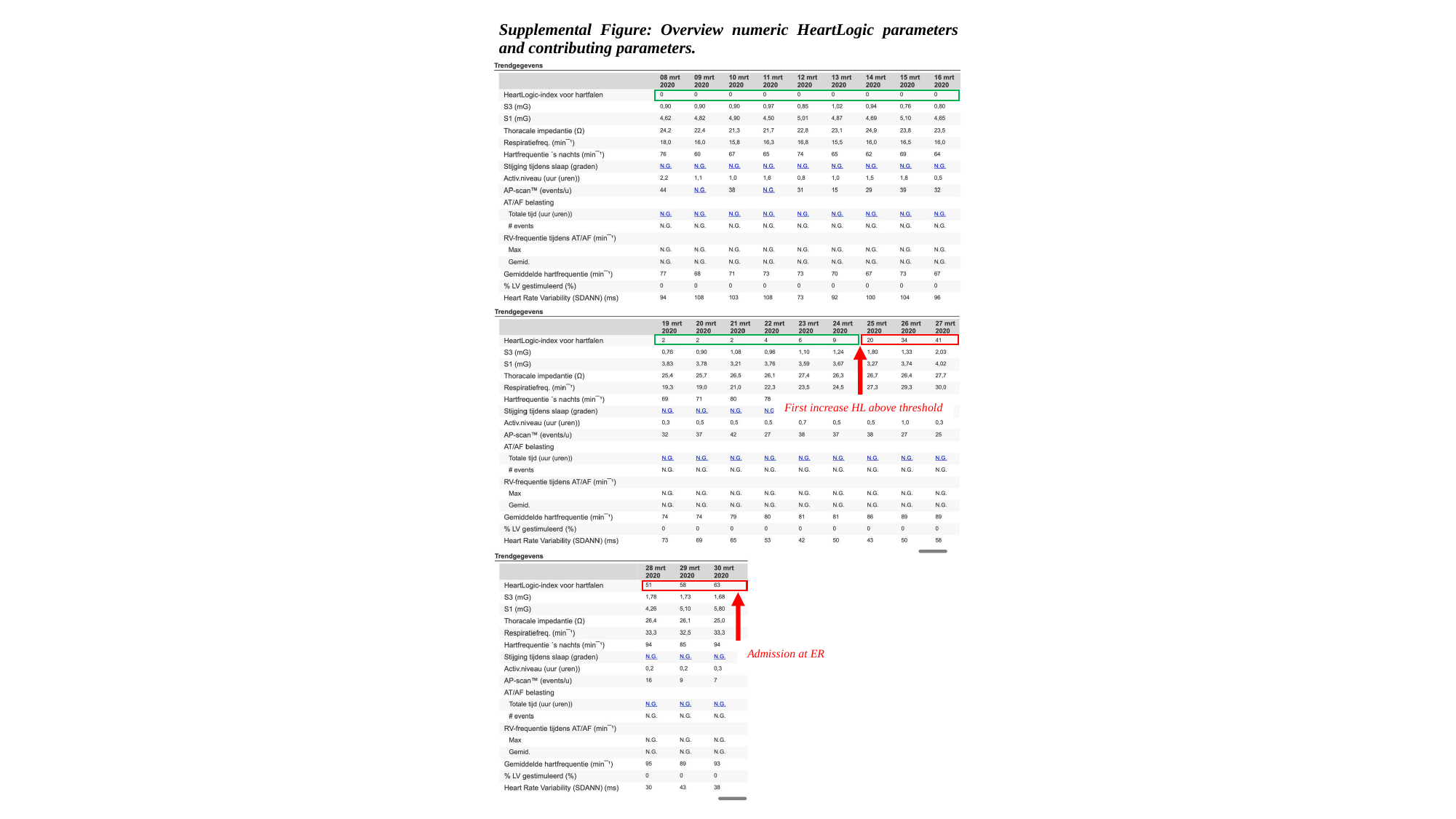

# Supplemental Figure: Overview numeric HeartLogic parameters and contributing parameters.
First increase HL above threshold
Admission at ER
